# 

**Published:** 1871-10

**Authors:** 


### EXCHANGES.

HERALD OF HEALTH, published by Wood & Holbrook, 12 and 15 Laight St., New York, at $2 per annum, is a first-class health journal.

---

THE MEDICAL TIMES.—The Philadelphia *Medical Times* is one of the most valuable exchanges that comes to our desk. It is published semi-monthly, by J. B. Lippincott & Co., at four dollars a year.

---

"GOOD HEALTH."—This popular health journal has the best list of contributors in the country. Its teachings are reliable and worth obeying, which is more than can be said of many so called health journals. Alex. Moore, Pub., Boston, Mass., at $2.00 a year.

---

"THE CHERUB," a paper for the people, devoted to literature, general information and the questions of the day, is a well printed and ably edited journal. A superb lithograph of Raphael's Cherub, and one year's subscription to the journal is offered for the small sum of sixty cents. Send for it—cheap enough !

---

THE MEDICAL WORLD, published by William Baldwin & Co., 21 Park Row, New York, and edited by Reuben A. Vance, M. D., is a valuable monthly medical journal, containing reports and communications from the principal medical writers of New York. Its price is only $1.50 a year. Very cheap and very good.

---

THE NORTHWESTERN MEDICAL AND SURGICAL JOURNAL, Vol. 2, No. 2, has been received. It is edited by Alexander J. Stone, M. D., and published by D. Ramaley, 28 Minnesota street, St. Paul, at $3.00 per annum. It is a voluminous monthly journal, containing the medical news of the great northwest. It deserves, and doubtless will receive, abundant patronage from the profession.

PETERSON—The cheapest and best Ladies' Magazine published in this country. Only $2.00 a year. Address Charles J. Peterson, 306 Chestnut St., Philadelphia, Pa.

INVENTOR AND MANUFACTURER, a monthly journal devoted to Science, Art, Manufacture, Inventions and Patents, is the title of a highly illustrated and ably edited journal, published at Cincinnati, Ohio, by T. Van Kannel & Co., at only *one dollar* a year. Send for it. It is worth more than twice that amount.

THE PACIFIC COAST JOURNAL, is the title of a neat little health journal published at San Francisco, Cal., by Carrie F. Young, M. D. We are one of those who think that women can do some things as well as men; and Mrs. Young demonstrates that editing a journal is one of them.

THE EYE IN HEALTH & DISEASE.—This is the title of a very useful, popular, hand-book upon the Eye. It is written by B. Joy Jeffries, M. D., and published by Alex. Moore, of Boston, at $1.50. Everybody, at some period in their lives, will have occasion to consult some one, relative to their eyes. By reading Dr. Jefferie's book, they can learn how to care for their own eyes, both in health and disease.

THE ALDINE.—This is the finest illustrated journal in this country. It is not made up of a series of cheap, sensational wood cuts, but contains choice engravings of permanent artistic value. Its typography is perfect, far surpassing anything of the kind in this country, while the literary matter is such as will be enjoyed by every intelligent household.

It is a large 16 page, monthly magazine, costing only $2.50 a year, including a handsome chromo.

James Sutton & Co., Publishers, 23 Liberty St., New York.

ACCORDING to recent estimates, the people of the United States pay $100,000,000 per year for medicines and medical services, exclusive of patent medicines, which adds to the sum $25,000,000 more.

IT IS said that the Internal Revenue Department has prepared a list, giving the name and post-office address of every one of the 49,798 physicians.

